# Regulatory, health technology assessment and company interactions: the current landscape and future ecosystem for drug development, review and reimbursement

**DOI:** 10.1017/S0266462323000144

**Published:** 2023-04-11

**Authors:** Ting Wang, Neil McAuslane, Wim G. Goettsch, Hubert G.M. Leufkens, Marie L. De Bruin

**Affiliations:** 1 Centre for Innovation in Regulatory Science (CIRS), London, UK; 2 Division of Pharmacoepidemiology and Clinical Pharmacology, Utrecht Institute for Pharmaceutical Sciences, Utrecht University, Utrecht, The Netherlands; 3 National Health Care Institute, Diemen, The Netherlands

**Keywords:** Health technology assessment, Early scientific advice, Stakeholder interactions, Collaboration, Evidentiary requirement

## Abstract

**Background:**

Multi-stakeholder interactions have evolved at product and policy levels. There is a need to assess the current and future landscape of interactions between companies, and regulatory and HTA agencies to address challenges and identify areas for improvement.

**Objectives:**

The aims of this study were to review the current interactions within and across regulatory and HTA agencies, and companies’ experiences in engaging in these activities; to assess the added value of interactions as well as limitations; to explore the future ecosystem for stakeholder interactions.

**Method:**

Three separate questionnaires were developed for companies, regulators and HTA agencies, respectively, to assess their experiences and perceptions. The responses were analyzed using descriptive statistics and discussed at a multi-stakeholder workshop. Key outcomes from the surveys and workshop discussion were reported.

**Results:**

All seven regulators and seven HTA agencies in the survey indicated that they had stakeholder interactions. More formal collaboration occurred with regulators compared with HTA agencies. All nine companies have taken early advice but indicated the need for future prioritization. Success indicators can be built at the product and therapy levels, with the added value of faster patient access. Four principles were proposed for the future ecosystem: separate remit and functions between regulators and HTA; align processes; converge evidence requirements where possible; increase transparency.

**Conclusions:**

This research brought together regulators, HTA agencies, companies to examine how they interact with one another. We propose measures of value and make recommendations on future evolution to enable better evidence generation and improve regulatory and HTA decision-making.

## Introduction

The process of bringing new medicines to market involves multiple stakeholders: pharmaceutical companies as the developer, regulators to grant market authorization based on quality, safety and efficacy, and health technology assessment (HTA) agencies to provide the recommendation for reimbursement. The patient’s role in the development, regulation and HTA processes for new medicines also continues to grow in importance to all decision makers, as the ultimate aim of all stakeholders in healthcare systems is to provide innovative medicine to patients in a timely and financially sustainable manner.

Nevertheless, the remit of regulators and HTA agencies may not fully align. Regulators aim to improve their pathways to provide a flexible mechanism for faster market authorization. HTA agencies and payers are under pressure to recommend reimbursement for new medicines within the constraint of the healthcare budget. In turn, companies need to generate evidence during development to ensure the product is approvable as well as reimbursable (1–3). Realizing the challenges and potential delay in patient access, stakeholders have started to work collaboratively to improve the efficiency of the decision-making process.

Over the last decade, regulatory and HTA interactions, as well as multi-HTA and multi-regulatory interactions, have evolved in theory and in practice. This has occurred at a product level as well as at a policy level, and spanned both national and cross-jurisdictional systems. Regulators have a long history of collaboration. Since its initiation in the 1990s, the International Council for Harmonization of Technical Requirements for Pharmaceuticals for Human Use (ICH) has been bringing together regulators and companies to develop harmonized guidelines that help to ensure that evidence submitted to regulators is presented in a consistent manner (4). For maturing regulatory agencies, reliance models have been put in place to facilitate the efficiency of the review process (5;6). For mature regulatory agencies, collaborative initiatives have been set up, such as Project Orbis for concurrent submission and review of oncology products (7) and the Access Consortium for medium-sized agencies to reduce duplication and align regulatory requirements (8). For HTA agencies, networks have been established to enable capacity building and shared learning, such as HTA international (HTAi) and The International Network of Agencies for Health Technology Assessment (INAHTA) at the global level, and HTAsiaLink and Health Technology Assessment Network of the Americas (RedETSA) at the regional level (9–11). Within Europe, the European Network for Health Technology Assessment (EUnetHTA) has been established to create an effective and sustainable network for HTA (12;13). Agencies also actively engage with companies to provide scientific advice to facilitate evidence generation during development. This advice comes either from the regulator, from the HTA agency, or jointly from both stakeholders (14–17). More recently, it has been suggested that scientific advice should expand from development to post-licensing evidence generation (PLEG) for life-cycle data collection (18).

Responding to the abundance of various stakeholder interactions, research has been undertaken to assess the learnings of these activities. Most studies to date focused on early scientific advice in terms of processes, discussion content and potential impact (15;16;19;20). A recent study by Ofori-Asenso et al. (21) examined the interactions between regulatory and HTA agencies and identified areas for further collaboration, such as early tripartite advice, parallel submission, adaptive licensing and PLEG. More recently, these channels of communication and the networks for interactions have been tested by the coronavirus disease-2019 (COVID-19) pandemic, illuminating both challenges and opportunities as new and repurposed medicines are developed and their assessment accelerated (22;23). Regulators, HTA agencies/payer agencies, pharmaceutical companies, patients and clinicians have a large influence on the development, approval and access to new medicines. Therefore, there is a need to identify not only the current but also the future landscape of stakeholder interactions. Our research focuses on the interactions among companies, regulators and HTA agencies to address challenges and examine potential solutions for the evolution of these interactions. This paper is based on the outcomes of a multi-stakeholder survey and workshop with the aim of identifying the current landscape and future ecosystem of stakeholder interactions to support drug development and patient access.

## Objectives and methods

### Survey

Centre for Innovation in Regulatory Science (CIRS) conducted a multi-stakeholder survey in February 2021 with the main objectives being to:

- Identify the current landscape of interactions within and across regulatory and HTA agencies, as well as companies’ experiences in engaging in these activities.
- Assess the added value of these interactions from each stakeholder’s perspective and identify how to measure success.
- Explore what the future ecosystem could be for interactions across stakeholders.

Three separate questionnaires were developed for companies, regulators and HTA agencies, respectively (Supplementary Materials 1–3). The pilot surveys were developed in January 2021 by the first author and were reviewed by all the coauthors with the purpose being to validate the clarity, format and applicability of the surveys. Feedback provided by coauthors was used to refine the wording of questions and to finalize the surveys on 3 February 2021. The questionnaires were distributed via email on 4 February 2021 to invited participants, who were asked to complete the questionnaire by 25 February 2021. A reminder email was sent on 22 February 2021 for returning the survey. The agency surveys were sent to CIRS contacts holding senior positions within 17 regulatory agencies and 15 HTA agencies in Australia, Canada, Europe and Asia. The agencies selected were either considered major international regulators/HTA agencies, or had been invited to the workshop. The agency surveys were made up of four multiple-choice, closed questions and three open-ended questions. The surveys focused on three sections: assessing the current interactions with different stakeholders; identifying the characteristics of an effective interaction model; and recommending an effective model for future interaction.

The company questionnaire was sent to senior management at 19 international pharmaceutical companies that were members of CIRS to ensure timeliness of the study and to maximize the response rate. The company survey consisted of six multiple-choice closed questions and three open-ended questions that focused on current interactions between stakeholders. The survey was composed of four sections: effective models of stakeholder interactions; convergence through interactions; focus on 2030 and improving the ecosystem for interactions; and ensuring interactions between different stakeholders are adding value. The company, regulator and HTA agency questionnaires contained analogous questions where appropriate. A free-text comment option was provided for each question to allow further clarification or comments.

### Workshop

A multi-stakeholder workshop was held virtually on 10–11 March 2021 on the topic of “Regulatory, HTA and payer interactions and collaborations: optimizing their use and outcome success” (24). The objectives of the workshop included to:

- Identify through case studies the key areas, types of interactions and collaborations between stakeholders that are effective, as well as the challenges and opportunities.
- Understand the value-add these interactions and collaborations bring to enabling improved decision-making by the stakeholders as well as how to address divergences and limitations.
- Make recommendations on what can be learnt across jurisdictions from the current initiatives so as to inform the future evolution of stakeholder interactions and collaborations to enable better evidence generation as well as improved outcomes for patient access.

Ninety-two senior representatives from regulatory agencies, industry, payers, HTA bodies, patient organizations, healthcare and academia participated in the workshop (the list of participating organizations is provided as Supplementary Material 4). The results from the survey were presented at the meeting, followed by keynote speakers, case studies and panel discussion. Participants were then arranged into four breakout groups. Each group was designed such that there was an even distribution of participants from each stakeholder type, with individual participants being selected randomly for any given group. The breakout topics were aligned with the survey topics and each breakout group was led by a chairperson selected by CIRS based on their expertise. A rapporteur for each group was also selected to document the discussion and present a summary of the discussion back to all workshop participants. This paper focused on the discussion output from the breakout groups.

### Data processing and analysis

The responses from the survey were tabulated into an Excel file manually and analyzed using descriptive statistics. Data were calculated as the absolute number of responses if respondents were less than 10, and the percentage of total responses if respondents were 10 or more. Ranking was applied where suitable. The first author conducted content analysis for free-text comments and open questions to identify key themes, before employing the constant comparative method. The results were reviewed by the second author to verify the phases and themes expressed by the study participants. The results for the breakout discussions were summarized by the first author based on the rapporteur presentations, as well as meeting recordings.

## Results

### Survey results

Representatives of seven (41 percent response rate) regulatory agencies and 7 HTA agencies (47 percent response rate) responded to the survey, which included key stakeholders from a mix of geographical locations. The regulatory agencies were Health Canada, the European Medicines Agency (EMA), Sweden’s Medical Products Agency (MPA), Switzerland’s Swissmedic, the Netherlands’ Medicines Evaluation Board (MEB), Singapore’s Health Sciences Authority (HSA) and China’s Center for Drug Evaluation (CDE). The responding HTA agencies were Australia’s Pharmaceutical Benefits Advisory Committee (PBAC), the Canadian Agency for Drugs and Technology in Health (CADTH), England’s National Institute for Health and Care Excellence (NICE), Sweden’s Tandvårds-Och Läkemedelsförmånsverket (TLV), China’s National Health Development Research Center, Singapore’s Agency for Care Effectiveness (ACE) and Thailand’s Health Intervention and Technology Assessment Program (HITAP). Nine out of the 19 pharmaceutical companies completed the survey (47 percent response rate). These companies were in the top 25 companies by R&D expenditure in 2019 (25), reflecting the research intensity of the companies and the innovativeness of their development pipelines.

#### Agencies’ experiences and perception of value of stakeholder interactions

**Figure 1.**
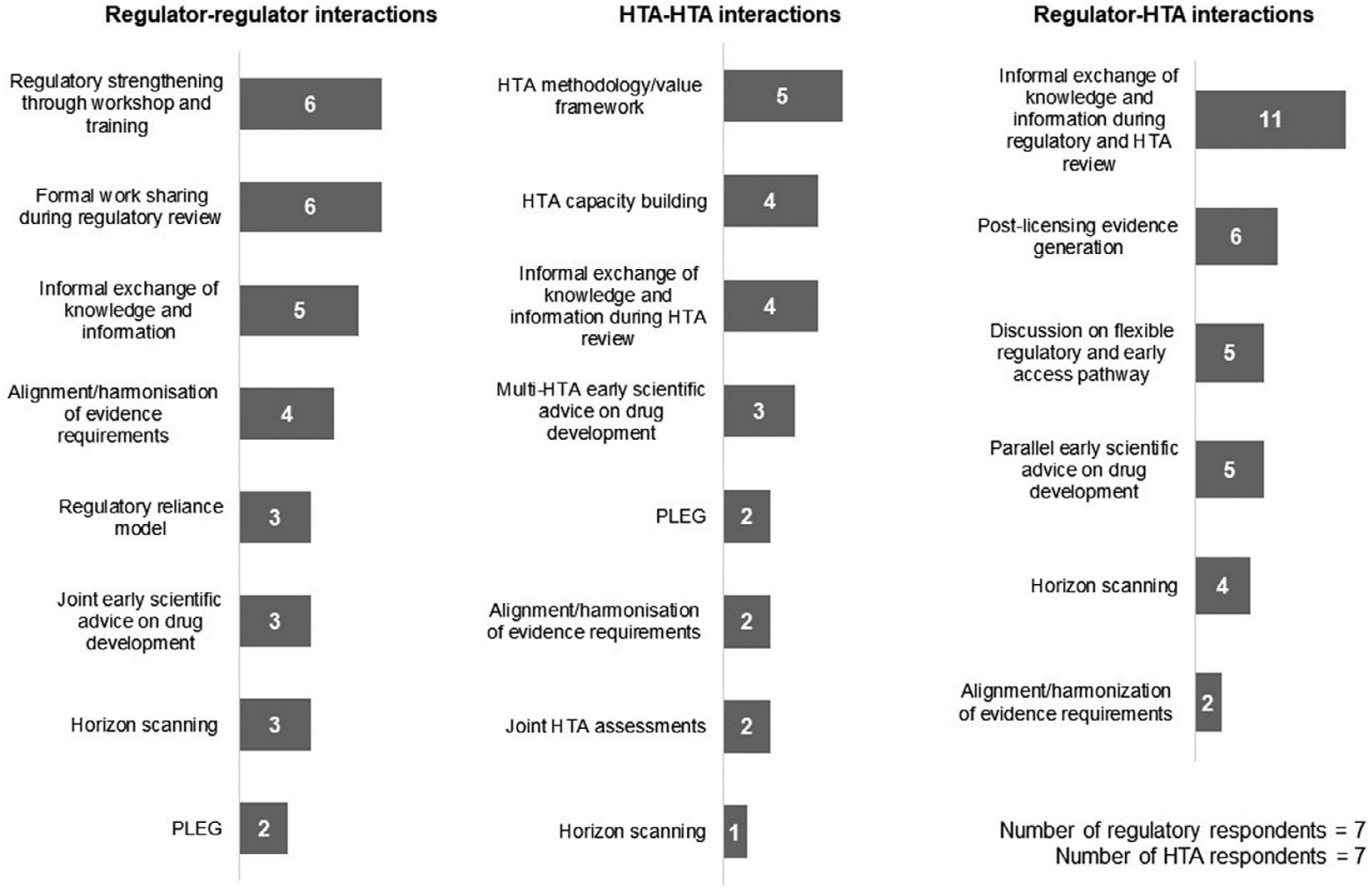
Overview of current interactions between agencies. HTA, health technology assessment; PLEG, post-licensing evidence generation.

All participating agencies indicated that they have interactions with other agencies. For regulatory–regulatory interactions, the top areas of interaction were formal work-sharing during review, regulatory strengthening through workshops, and training and informal exchange of knowledge and information. Respondents saw value in reducing duplication of work and providing an opportunity for capacity building, enabling more efficient drug development and support for post-approval activities. For HTA–HTA interactions, the top areas of interaction focused on HTA methodology/framework, HTA capacity building, and informal exchange of knowledge and information. These interactions were reported as being useful to improve understanding of the divergences in evidence requirements and to validate agency internal thinking (Figure 1). Two European HTA respondents were experienced in joint assessment through EUnetHTA.

For cross-stakeholder interactions, the top areas of regulatory–HTA interaction were the exchange of knowledge and information during regulatory and HTA review (85 percent of total respondents) and PLEG (46 percent of total respondents). Only 2 of 14 agencies reported on alignment/harmonization of evidence requirements. Regulatory–HTA interactions were seen to have fewer practical advantages but provided the opportunity to learn about the complexity of different systems. Both regulators and HTA agencies reported having interactions with payers to facilitate the informal exchange of knowledge and information. HTA–payer interactions primarily focused on the implementation of HTA recommendations, discussion of pricing and budget impact, as well as discussion of conditional reimbursement/managed entry schemes.

#### Companies’ experiences and perceptions of value of stakeholder interactions

All nine companies reported having experiences in seeking early scientific advice with a regulator, HTA agency or through parallel regulatory–HTA advice. Five companies had experience with multi-HTA joint advice and four with joint multi-regulator advice. Advice on PLEG plans tended to be more common with regulators than with HTA agencies (five vs. two companies). Companies indicated that this interaction should be prioritized for products responding to unmet medical need, or new technologies such as cell/gene therapies. Companies also had interactions through public–private partnerships such as Get-Real-Initiatives to facilitate alignment of evidence requirements (eight respondents), as well as input into evidence standards at the policy level (seven respondents).

**Figure 2.**
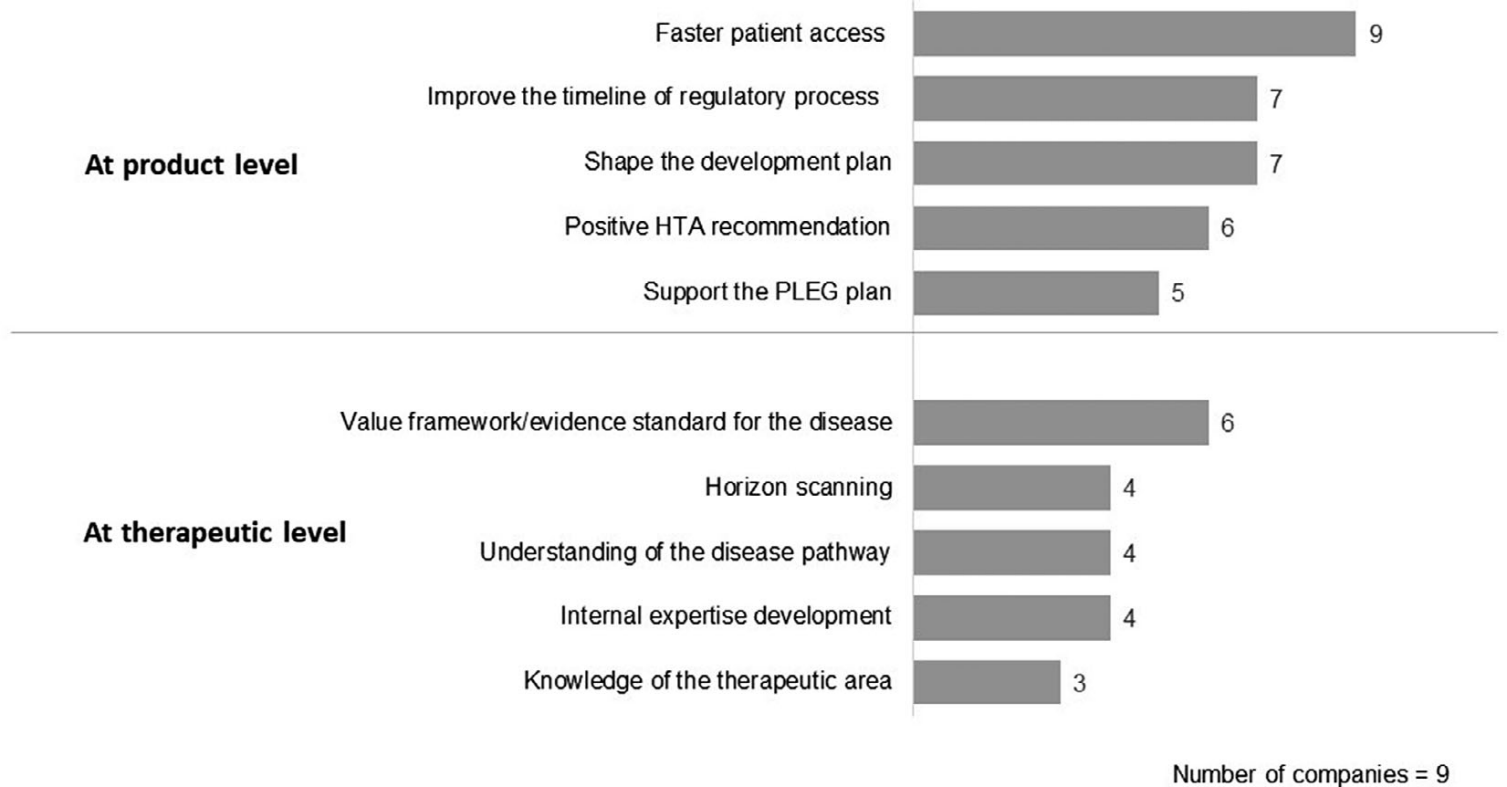
Companies’ perspectives on indicators to measure the value of stakeholder interactions. HTA, health technology assessment; PLEG, post-licensing evidence generation.

Six companies reported that external interactions were a priority and that there were plans for future engagement, while three companies had agreed on this in principle, but were subject to the resource available to support these interactions. Six companies indicated that the “success of interactions are measured subjectively” with a partially developed set of indicators, while three companies did not have any indicators in place to measure external interactions. All companies responded on the key areas that potential success indicators could be built on at both the product and therapy level (Figure 2). At the policy level, the value of stakeholder interactions could be measured by “input into guideline development”, promoting “good HTA review practice”, supporting “HTA capacity building” and “Regulatory strengthening.”

#### Effective model of current interaction between regulators, HTA agencies and companies

Respondents noted that interactions were effective if the outcome aligned with the aim of the activities. ICH was rated by both companies and agencies as an effective model to support the harmonization of technical requirements. EUnetHTA early scientific advice was voted as an effective collaboration to support evidence generation. Access Consortium and Orbis projects were selected as an effective way of formal regulatory work sharing, while the Medicines Evaluation Board (MEB) and the National Health Care Institute (ZIN) parallel process in the Netherlands and the Innovative Licensing and Access Pathway in the UK were viewed as good models to align regulatory and HTA process. With regard to improving agency decision-making, international advisory committee and international collaboration programs were seen as effective, while national regulatory and HTA informal information exchange were recommended to enable process efficiency.

#### Future ecosystem for interaction between regulators, HTA agencies and companies

When asked about the ideal ecosystem for multi-stakeholder interactions in the future, four key principles emerged from the responses:

1. Separate remit and functions of the regulator and HTA agency: to acknowledge and provide clarification on scope and remit between regulators and HTA agencies while increasing mutual understanding between the two stakeholders.
2. Convergences of evidence: develop common methodology and evidence standards where possible, so that drug development is primed to meet both regulatory and HTA requirements.
3. Align process and use reliance: where appropriate, further align regulatory and HTA processes with formal and/or informal information exchange to ensure process efficiency, advance reliance mechanisms for regulators and enhance collaboration among HTA agencies such as work sharing or leveraging other agencies’ work.
4. Transparency: increase trust between multiple stakeholders and propose a transparency agreement for information sharing. At the jurisdictional level, there should be collaborative approaches to horizon scanning to support innovation and facilitate patient access.

### Workshop breakout groups

**Figure 3.**
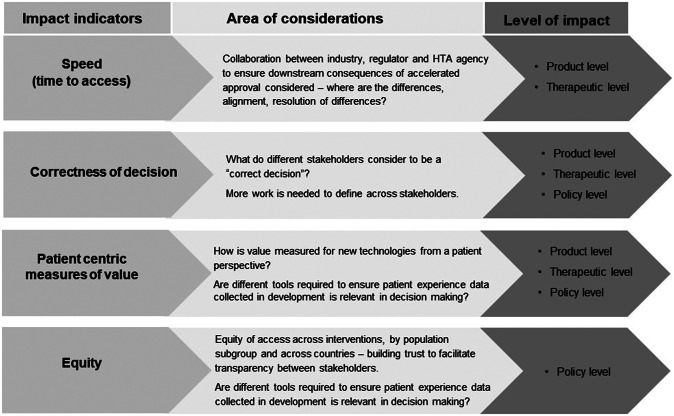
Recommended indicators to measure the impact of stakeholder interactions. HTA, health technology assessment.

Details of workshop presentations, case studies and panel discussions have been published (24). This paper focuses on the breakout discussions during the workshop. The discussion participants reviewed the survey results and reflected on their own experiences of stakeholder interactions. EUnetHTA parallel advice was reported to promote cross-functional collaboration within companies and among agencies. Nevertheless, challenges were identified by participants, for example, companies need to achieve consensus on the evidence-generation plan between internal regulatory and HTA functions; companies may assume that not following the scientific advice will impact the HTA recommendation; there is a lack of consensus on post-licensing data sharing between regulatory and HTA agencies; and multiple data sources can be an issue. Participants emphasized the evidence needs for comparative effectiveness post-approval and suggested that HTA agencies and payers align on affordability. Four success indicators to measure interactions were recommended: speed (time to patient access), “correctness” of decisions (subject to each stakeholder’s perspective), patient relevance of the evidence generated and equity of access (Figure 3). However, participants noted that measures should not be unidimensional; the speed to patient access cannot be compromised by the quality of decision-making. It was suggested that the correctness of decisions needs to balance with the speed of decision-making, though different stakeholders held different views on the nature of the trade-off between quality and speed. Further research is needed to understand and define these indicators. Agencies indicated that the intangible aspects of interactions were important, such as building relationships and trust with their peer agencies, and improving knowledge of a new technology, which were difficult to measure quantitatively. It was suggested to assess the change of decision-making behaviors of stakeholders as a consequence of interactions.

**Figure 4.**
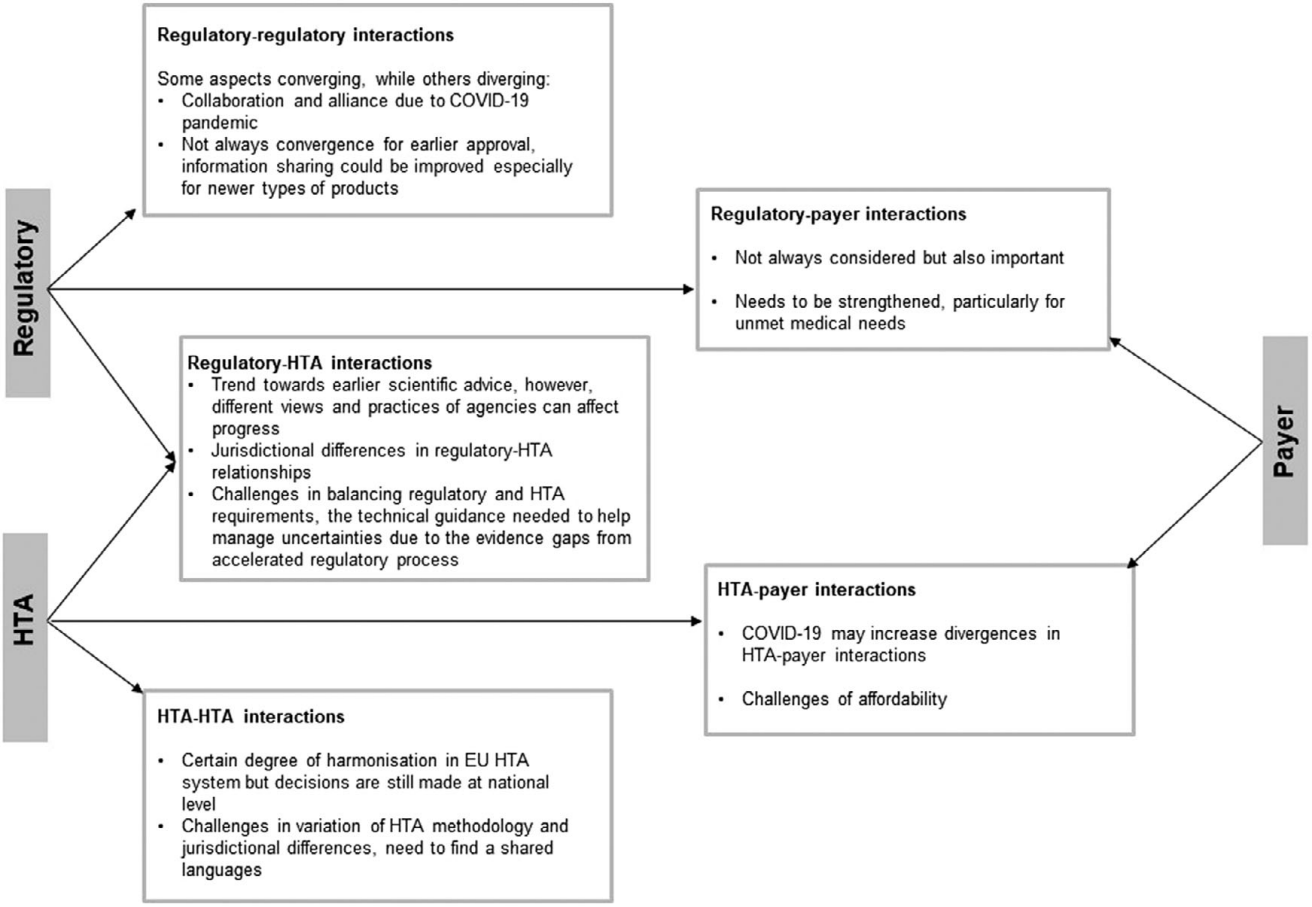
Direction of evolution of interactions between different stakeholders. EU, European union; HTA, health technology assessment.

Finally, the breakout group participants reviewed different types of stakeholder interaction and their future evolution (Figure 4). They also considered the impact of the COVID-19 pandemic, which has changed ways of working and accelerated the decision-making process. There was a concern that “vaccine nationalism” may reverse this and potentially lead to more divergence among jurisdictions. The participants illuminated the future ecosystem for interactions. During drug development, stakeholders would have shared language to agree on the unmet need, clinical effectiveness, uncertainty and methodology; a stable platform for early dialogue that would enable alignment at the start of process, and strengthen networks to help foster valuable collaborations. During the post-licensing stage, there would be clear requirements and standards for post-approval data collection and better use of historical control data. Participants also suggested that further interaction could take the form of an informal network that may focus on public health-related or policy-related topics.

## Discussion

Over the past decade, interactions between regulators and HTA agencies, as well as multi-regulator and multi-HTA interactions, have taken place to better support companies on clinical development, align the decision-making process among agencies to encourage efficiency and better-informed decision-making, and promote trust and reliance between all stakeholders (21;26;27). This multi-stakeholder survey and workshop assessed the current landscape of multi-stakeholder interactions, their added value and the future development of these activities.

The survey illustrated different levels of interactions, particularly more formal work sharing between regulators compared with the informal exchange of information among HTA agencies. This may relate to the longer history of regulatory agencies compared with the formal initialization of HTA, which has allowed mechanisms to be tested, and trust to be built. Formal processes such as reliance models and standardized technical requirements through ICH fostered collaboration between regulators (26;28). EUnetHTA has provided the platform to test multi-HTA collaboration, which led to the formal production of joint clinical assessment (JCA) to be fully implemented by 2029 (29). It is, however, critical for stakeholders in member states to collaborate in coming years to ensure that JCA will be used effectively in local decision-making, rather than being a duplicative process. Our study also identified the appetite for HTA agencies to learn from the collaborative models of regulators, such as the Orbis project, to expand collaboration outside Europe. We note that since the study was conducted, 6 HTA agencies from Australia, Canada and the UK agencies started to explore the possibility of work sharing to improve efficiency (30). To achieve this goal, capacity building and alignment in the HTA methodology/framework will be important; these two areas were rated as the top areas of focus by HTA respondents in the study.

The most important measure identified by companies was faster patient access. HTA-regulatory procedural and timeline alignment is viewed as a valuable interaction. For example, parallel HTA and regulatory reviews available in Australia and Canada have shortened the time to HTA decision/recommendation and by extension have led to quicker patient access (31). In the Netherlands, a pilot was launched in 2019 for a parallel process with formal coordination between MEB and ZIN. A recent example for Astellas’ roxadustat showed that the parallel process allowed ZIN to rule on the reimbursement immediately after registration (32). The successful pilot demonstrated a time saving of 3 months and has moved into a more structural collaboration. The Dutch model provided learnings for future national regulatory and HTA collaboration. Our findings acknowledged that regulators and HTA agencies should remain separate in function and remit, but more work could be done to converge evidence requirements where possible. For example, palbociclib was approved by EMA in 2016 for the treatment of breast cancer. However, the uncertainty due to lack of evidence on overall survival and treatment length led to divergent HTA recommendations in Europe. To investigate the evidence gap for palbociclib, a EUnetHTA PLEG pilot was conducted in 2021; this interaction identified common research recommendations among participating agencies, and saw the opportunity for collaboration between HTA agencies using cross-nationwide real-world evidence (RWE) to facilitate the initial HTA decision and subsequent reassessment (33).

Early scientific advice developed in recent years supported the development and PLEG for companies, facilitated conversations among agencies and enabled better understanding between stakeholders. Nevertheless, these activities are resource-consuming, and the workshop participants raised the question of the capacity of companies and agencies to participate in such activities. This in turn requires prioritization. EUnetHTA joint scientific consultation listed its essential criteria: unmet medical needs; first in class; potential impact on patients/public health; significant cross-border dimension; major union-wide added value or research priorities; and breakthrough technology for oncology products and/or advanced therapy medicinal products (34). The criteria ensured that the resources from agencies were prioritized, in particular for interactions involving multiple agencies. These principles are mirrored with companies’ priorities, as noted in our survey results. Studies on aligning each stakeholder’s definition on unmet medical need contributed to mutual understanding of stakeholders’ priorities (18;35).

Planning for early advice is also key; this needs to be early enough to shape the development plan, but not too early in order to ensure that sufficient evidence has been generated to support a meaningful dialogue. Therefore, future improvement should focus on clarifying the optimal timing to seek advice from regulators and HTA agencies. Our research suggested that the interaction should not be a one-off activity but allow for a more flexible and iterative process for advice, especially considering the life cycle approach to collect data for medicines’ review and reimbursement. In addition, early advice could be more transparent in a later stage of life-cycle decision-making. Operational actions were suggested to improve efficiency, including consolidating learnings from scientific advice and speeding up administration steps. The current early advice activities are mainly provided by established HTA agencies in Europe and Canada, which are important markets for companies from a commercial perspective. As HTA systems are continuously developing and improving in other jurisdictions, agencies could consider further stakeholder interactions, such as providing advice to companies on the development plan and local submission. Companies should in turn decide strategically when and which agencies to seek advice from. Further research can be conducted to investigate the stakeholder interactions within emerging markets. We also saw an opportunity for informal networks to complement formal advice and contribute to not only product-related topics but also policy and public health-related discussions.

Stakeholder interactions were seen as critical and beneficial for future drug development and availability; the workshop breakout groups pictured the ideal future ecosystem. However, the agility of regulatory and HTA systems have been tested during the COVID-19 pandemic. Researchers have analyzed potential scenarios for the future of medicines and social policy in 2030; increased knowledge sharing, trust and openness in science, as well as partnership have been identified as key drivers for sustainable flow and transformative healing scenarios (36). The optimal direction of travel requires further dialogue, interaction and trust among stakeholders. Suggestions were proposed to improve current experiences, such as patient centricity, sharing common objectives among stakeholders and establishing a stable platform for continuous dialogue. To move from identifying divergence to enabling more convergence, the breakout groups suggested more work-sharing and reliance models between regulators, alignment on affordability between HTA agencies and payers, and increased transparency of PLEG requirements between regulators and HTA agencies.

Our research identified four potential areas to measure value: time to access, correctness of decision, and patient-centric measures of value and equity. Findings from this study will contribute to further discussions on building good practice into stakeholder interactions. An immediate next step can be a study to develop performance metrics to measure the value of interactions from the perspectives of regulators, HTA agencies and companies. Apart from potential quantitative indicators, the participants also raised the qualitative value of interacting with other stakeholders, such as learning about new technology, validating internal thinking and building trust with (and improving understanding of) other agencies. An interesting suggestion for further discussion was the possibility to assess behavior changes in decision-making following these interactions.

## Study limitation

This study addressed the key components of stakeholder interactions from the regulator, HTA agency and company perspective. Its limitation is the lack of patient and payer feedback in the survey. Nevertheless, patient representatives and payer organizations were present at the workshop, which added their voice to the overall discussion and development of suggestions. Another limitation is the number of survey respondents, which, due to the study time frame, was limited to seven regulators, seven HTA agencies and nine companies. However, this is complemented by the larger number of participants at the workshop, which provided further insights on the topics addressed in the survey.

## Conclusions

The multi-stakeholder interactions among regulators and HTA agencies, as well as between regulators and HTA agencies, are important for ensuring a more efficient process from development to patient access. The outcome of the survey and workshop identified current interactions, challenges and gaps, and suggested indicators that could be built to measure the value of interactions. This research also assessed perceptions of the future evolution of these activities. Four key principles were identified for further development of interactions: keep the remit and functions of stakeholders separate; align processes; converge evidence requirements when it is scientifically justifiable; and increase transparency to build trust.

## Supplementary material

For supplementary material accompanying this paper visit https://doi.org/10.1017/S0266462323000144. click here to view supplementary material
