# Supplementary material for: Regulatory, health technology assessment and company interactions: the current landscape and future ecosystem for drug development, review and reimbursement
Source: Int J Technol Assess Health Care. 2023 Apr 11;39(1):e20. doi: 10.1017/S0266462323000144 (PMC11574548; doi:10.1017/S0266462323000144)
Supplement: Supplementary file 1 [file S0266462323000144sup.zip › S0266462323000144sup001.docx]

**2021 Centre for Innovation in Regulatory Science (CIRS) focus survey**

**Section 1: Effective models of engagement**

1. **Does your company utilize any interaction/collaboration with other stakeholders to support evidence generation? Please select all that applies from the options on below**

Early scientific advice during drug development from a regulatory agency

Joint early scientific advice on drug development from multiple regulatory agencies

Early scientific advice during drug development from an HTA agency

Early scientific advice during drug development from multiple HTA agencies

Parallel Early scientific advice during drug development given from regulatory and HTA agencies

Interaction with regulatory agencies on the post licensing evidence generation plan (PLEG)

Interaction with HTA agencies on the post licensing evidence generation plan (PLEG)

Others, please specify __________________________

1. **Is your company involved in any interaction/collaboration with other stakeholders regarding alignment/harmonization on evidence standard? Please select all that applies**

Harmonization evidence requirements for regulatory agencies (eg. ICH)

Standardized evidence requirements by HTA agencies (eg. EUnetHTA core model)

Public-private partnership/ topic driven taskforce on evidence requirements, such as Real-World Evidence

Input into evidence standard at policy level (eg, responses to agencies’ public consultation guidelines)

Others, please specify __________________________

**3a) For each purpose of interaction/collaboration across stakeholders, please provide an example that your company perceives as an effective model of engagement and the rationale of your selection.**

| **Purpose of the interaction** | **An example of an effective interaction/collaboration** | **The reason why this is an effective model** |
| --- | --- | --- |
| To support evidence generation during development |  |  |
| To support evidence generation during post-approval |  |  |
| To align/ harmonize evidence standard |  |  |

**3b) For each purpose of interaction/collaboration across stakeholders, please comments on the main challenges you perceive and what will be the potential solutions?**

| **Purpose of the interaction** | **Main challenges** | **Potential solutions** |
| --- | --- | --- |
| To support evidence generation during development |  |  |
| To support evidence generation during post-approval |  |  |
| To align/ harmonize evidence standard |  |  |

**Section 2: Convergence through collaboration**

1. **Does your company have a systematic process to decide which agencies to interact with during development, when to interact and for what products?**

Yes, fully integrated systematic decision-making process on stakeholder interactions

Yes, partial integrated approach with decisions made on ad hoc basis

No

Others, please specify____________

1. **In your opinion, what types of products will benefit the most from the stakeholder interactions/collaborations?**

| **Purpose of the interaction** | **Type of products that will benefit from the interaction** |
| --- | --- |
| To support evidence generation during development | All New Active Substances (NASs)  Products responding to rare disease  Products responding to chronic disease  Products responding to unmet medical need  New technology, such as Cell/gene therapy, ATMP  Repurposed medicine responding to healthcare urgency (eg. COVID-19)  Others |
| To support evidence generation during post-approval | All New Active Substances (NASs)  Products responding to rare disease  Products responding to chronic disease  Products responding to unmet medical need  New technology, such as Cell/gene therapy, ATMP  Repurposed medicine responding to healthcare urgency (eg. COVID-19)  Others |
| To align/ harmonize evidence standard | All New Active Substances (NASs)  Products responding to rare disease  Products responding to chronic disease  Products responding to unmet medical need  New technology, such as Cell/gene therapy, ATMP  Repurposed medicine responding to healthcare urgency (eg. COVID-19)  Others |

**Section 3: Focus on 2030 and what would an ideal ecosystem be for interactions and collaboration**

1. **In your company, is interaction/collaboration with stakeholders a priority in the strategic plan?**

Yes, external collaboration is a priority for my company and there are plans for future activities

Yes, in principle but it will be depending on the resource (financial, manpower, time etc)

No further plans beyond our current activities

There is a plan to reduce the number of interactions/collaborations

Please provide any comment you may have: ________

| 1. **Focus on 2030, what would you like to see as an ideal ecosystem for interactions and collaborations across stakeholders? eg. Separate, aligned, converged, harmonized, collaborative, reliant? And what are the building blocks that will enable such an evolution?**  \| **Expectation of the future ecosystem across regulatory, HTA, payer to support the development, review and access of new medicine** \| **Please provide an example of potential building blocks that will enable such an evolution** \| \| --- \| --- \| \|  \|  \| |
| --- | --- | --- | --- | --- |

**Section 4: Ensuring that interactions and collaborations between different stakeholders are adding value**

1. **In your company, is there a set of indicators developed to measure the success of stakeholder interactions/collaboration**

Yes, a set of formal indicators is in place. Please provide an example:

Partially, the success of interaction/collaboration is measured subjectively

No, no indicators in place

Others

Please provide a comment________

1. **In your opinion, what are the key areas that the success indicators could be built on? Please select top three for each level.**

| **Level** | **Key areas to build success indicators** |
| --- | --- |
| Product level | Shape the development plan  Support the PLEG plan  Improve the timeline of regulatory process  Positive HTA recommendation  Faster patient access  Other, please specify: |
| Therapeutic level | Internal expertise development  Knowledge on the therapeutic area  Understanding of the disease pathway  Horizon scanning  Value framework/evidence standard for the disease  Other, please specify: |
| Policy level | Input into guideline development  Regulatory strengthening  Best regulatory review practice  HTA capacity building  Good HTA review practice  Other, please specify: |
| Others |  |
