# Supplementary material for: Regulatory, health technology assessment and company interactions: the current landscape and future ecosystem for drug development, review and reimbursement
Source: Int J Technol Assess Health Care. 2023 Apr 11;39(1):e20. doi: 10.1017/S0266462323000144 (PMC11574548; doi:10.1017/S0266462323000144)
Supplement: Supplementary file 1 [file S0266462323000144sup.zip › S0266462323000144sup003.docx]

**2021 Centre for Innovation in Regulatory Science (CIRS) focus survey**

**Part A: Agency overview**

1. **Is your agency currently involved in any interaction/collaboration with other stakeholders? Please select all that applies from the options on below**

Yes, interaction/collaboration with a Regulatory agency (or agencies) (if yes, please go to question 2.1)

Yes, interaction/collaboration with another HTA agency (or agencies) (if yes, please go to question 2.2)

Yes, interaction/collaboration with a payer agency (or agencies) (if yes, please go to question 2.3)

Yes, involvement in public-private partnership/ topic driven taskforce (if yes, please go to question 2.4)

No - no involvement in any interactions/collaboration with other stakeholders (Please go to question 3)

Others, please specify:

1. **If “Yes”, please provide the areas of the interactions/collaborations**

**2.1 Current interaction/collaboration with a Regulatory agency (or agencies)** (please select all that apply)

Horizon scanning

Parallel early scientific advice on drug development

Informal exchange of knowledge and information during regulatory and HTA review

Discussion on flexible regulatory and early access pathway

Alignment/harmonisation of evidence requirements

Post-licensing evidence generation

Other, please specify:

**2.2 Current interaction/collaboration with another HTA agency (or agencies)** (please select all that apply)

Horizon scanning

Multi-HTA early scientific advice on drug development

Informal exchange of knowledge and information during HTA review

Joint HTA assessments

Alignment/harmonisation of evidence requirements

Post-licensing evidence generation

HTA methodology/value framework

HTA capacity building

Other, please specify:

- 1. **Current interaction/collaboration with a payer agency (or agencies)** (please select all that apply)

Horizon scanning

Parallel/joint early scientific advice on drug development

Informal exchange of knowledge and information

Alignment/harmonisation of evidence requirements

Discussion on conditional reimbursement/managed entry scheme

Pricing and budget impact

HTA recommendation implementation

Other, please specify:

- 1. **Current public-private collaboration/ topic driven taskforce** (free text)

| Please specify the key areas of collaboration |
| --- |

1. **Please provide information on any interactions/collaborations that were not covered above.**

|  |
| --- |

**Part B: Assessment of the key interactions**

1. **In your opinion, what are the added value of stakeholder interactions/collaborations for your agency? Please select all options that apply from the table below**

| **Aspect of added value of stakeholder interactions/collaboration for your agency** | **Regulatory and HTA interaction** | **HTA and HTA interaction** | **HTA and payer interaction** | **Public-private partnership/**  **Topic driven taskforce** |
| --- | --- | --- | --- | --- |
| Early signal to my agency on what is the areas of unmet needs and healthcare priorities |  |  |  |  |
| Provides insight into policy implications of emerging technologies and health threats |  |  |  |  |
| Enables a more effective and efficient drug development |  |  |  |  |
| Provides early insights into new innovative medicines prior to their assessment |  |  |  |  |
|  | | | | |
| Supports internal agency decisions at time of assessment |  |  |  |  |
| Reduce duplication of work |  |  |  |  |
| Improves the timing of the submission and review process |  |  |  |  |
| Supports future HTA decisions |  |  |  |  |
| Supports post-approval activities |  |  |  |  |
|  | | | | |
| Improves understanding of the divergences across evidentiary requirements |  |  |  |  |
| Validates internal thinking within my agency |  |  |  |  |
| Provides a learning opportunity about complexity of multiple system interactions |  |  |  |  |
| Provides an opportunity for capacity building and strengthening |  |  |  |  |
|  | | | | |
| If the aspect of added value of these interactions to your agency is not captured in the statements above, please provide the details here: |  |  |  |  |

1. **For each type of interaction/collaboration across different stakeholders, please provide an example that your agency perceives as an effective model of engagement and the rationale of your selection.**

| **Type** | **Name of the interaction/collaboration** | **The reason why this is an effective model** |
| --- | --- | --- |
| Regulatory and HTA interaction |  |  |
| HTA and HTA interaction |  |  |
| HTA and payer interaction |  |  |
| Public-private partnership |  |  |

**Part C: Future ecosystem for multi-stakeholder interactions**

1. **In your agency, is further interaction/collaboration with stakeholders a priority in the strategic plan?**

Yes, external collaboration is a priority for my agency and there are plans for future activities

Yes, in principle but it will be depending on the resource (financial, manpower, time etc)

No further plans beyond our current activities

There is a plan to reduce the number of interactions/collaborations

Please provide a comment________

| 1. **Focus on 2030, what would you like to see as an ideal ecosystem for interactions and collaborations across stakeholders? eg. Separate, aligned, converged, harmonized, collaborative, reliant? And what are the building blocks that will enable such an evolution?**  \| **Expectation of the future ecosystem across regulatory, HTA, payer to support the development, review and access of new medicine** \| **Please provide an example of potential building blocks that will enable such an evolution** \| \| --- \| --- \| \|  \|  \| |
| --- | --- | --- | --- | --- |
