# Supplementary material for: Regulatory, health technology assessment and company interactions: the current landscape and future ecosystem for drug development, review and reimbursement
Source: Int J Technol Assess Health Care. 2023 Apr 11;39(1):e20. doi: 10.1017/S0266462323000144 (PMC11574548; doi:10.1017/S0266462323000144)
Supplement: Supplementary file 1 [file S0266462323000144sup.zip › S0266462323000144sup004.docx]

| **Agency participants** |
| --- |
| Agency for Care Effectiveness (ACE), Ministry of Health, Singapore |
| AOK Health Insurance, Germany, MEDEV, Brussels |
| Canadian Agency for Drugs and Technologies in Health (CADTH), Canada |
| Center for Drug Evaluation (CDE), Chinese Taipei |
| Department of Health, Pharmaceutical Benefits Advisory Committee (PBAC), Australia |
| European Commission/DG SANTE, Belgium |
| European Medicines Agency (EMA), The Netherlands |
| Food and Drug Administration (FDA), USA |
| Federal Joint Committee (G-BA), Germany |
| GKV-Spitzenverband, National Association of Statutory Health Insurance Funds, Germany |
| Health Canada, Canada |
| Medicinal Products Agency (MPA), Sweden |
| Medicines Evaluation Board (MEB), The Netherlands |
| Medicines and Healthcare products Regulatory Agency (MHRA), UK |
| Ministry of Health, Israel |
| National Health Care Institute (ZIN), The Netherlands |
| National Institute for Clinical Excellence in Health and Social Services (INESSS), Canada |
| National Institute for Health and Care Excellence (NICE), UK |
| South African Health Products Regulatory Authority (SAHPRA), South Africa |
| Scottish Medicines Consortium (SMC), UK |
| Swiss Federal Office of Public Health, Switzerland |
| Swissmedic, Switzerland |
| Taiwan Food and Drug Administration (TFDA), Chinese Taipei |
| Therapeutic Goods Administration (TGA), Australia |
| The Dental and Pharmaceutical Benefits Agency (TLV), Sweden |
| Turkish Medicines and Medical Devices Agency (TMMDA), Turkey |
| **Company participants** |
| Abbvie |
| Amgen |
| Astellas |
| AstraZeneca |
| Bayer |
| Biogen |
| CSL Behring |
| Eisai |
| Eli Lilly |
| F. Hoffmann-La Roche |
| GlaxoSmithKline |
| H Lundbeck |
| Ipsen |
| Janssen Pharmaceuticals |
| LEO Pharma |
| Lundbeck A/S |
| MSD |
| Novartis |
| Pfizer |
| Sanofi |
| Takeda |
| **Other participants** |
| Bill and Melinda Gates Foundation, UK |
| Center for the Evaluation of Value & Risk in Health, Tufts Medical Center, USA |
| Centre of Regulatory Excellence, Singapore |
| Consilium Salmonson & Hemmings, Sweden |
| Critical Path Institute, USA |
| Danish Centre for Health Economics, Faculty of Health Sciences, University of Southern Denmark |
| Golden Jubilee National Hospital, UK |
| Office of Health Economics, UK |
| PharmaExec Consulting AB, Sweden |
| University of Adelaide, Australia |
| Utrecht University, The Netherlands |
